# Attitudes of Healthcare Workers in Israel towards the Fourth Dose of COVID-19 Vaccine

**DOI:** 10.3390/vaccines11020385

**Published:** 2023-02-07

**Authors:** Shira Ramot, Orna Tal

**Affiliations:** 1 Department of Management, Health Systems Management Program, Bar Ilan University, Ramat Gan 5290002, Israel; 2 Shamir Medical Center (Assaf Harofeh), Zerifin 7033001, Israel; 3 ICET—Israeli Center for Emerging Technologies, Zerifin 7033001, Israel

**Keywords:** COVID-19, booster dose, healthcare workers, hesitancy, risk–benefit perception, vaccines

## Abstract

Attitudes of healthcare workers (HCWs) toward vaccines are extremely important for increasing vaccination coverage. We conducted a cross-sectional study at the beginning of the fourth COVID-19 vaccination dose campaign among 124 HCWs to evaluate attitudes towards the fourth dose and willingness to get vaccinated. At that time, Israel was the first country to approve the fourth vaccine dose. Most women were unwilling to get the fourth vaccine dose compared to men; 53.9% of physicians were unwilling to get vaccinated compared to 83.3% of nurses and 69% of other HCWs professions. The most frequent concerns regarding the vaccine were its efficacy, benefit, and necessity. The perceived risk and perceived severity of the health risk involved with getting vaccinated with the fourth dose were higher among HCWs who stated that they would not get vaccinated compared to those who were vaccinated or intended to get vaccinated. In contrast, HCWs who were vaccinated with the fourth dose, or those who were planning to get vaccinated, gave higher scores to the perceived benefit of the booster, its advantages, its perceived safety, its ability to protect from severe illness, and the perceived extent of scientific information about the risk associated with the booster. A logistic regression model revealed that perception of the fourth dose’s benefits and risk significantly predict the willingness of HCWs to get vaccinated. Willingness to vaccinate their own children, acceptance of a hypothetical annual booster vaccine, and having less severe adverse effects after prior vaccination were also associated with willingness to get the fourth dose. These findings could help policy makers in developing strategies to expand the acceptance and coverage of the COVID-19 booster doses.

## 1. Introduction

Israel was the first country to approve the fourth dose of the coronavirus disease 2019 (COVID-19) mRNA vaccine. This approval, concomitantly with the emergence of the Omicron (B.1.1.529) variant of severe acute respiratory syndrome coronavirus-2 (SARS-CoV-2), has brought to attention questions about vaccination policies and the balance among governmental accountability towards public health, the decreased virulence of SARS-CoV-2, and the scarcity of real-world evidence about the efficacy and safety of the fourth dose of the vaccine [1]. All these factors may affect the willingness of healthcare workers (HCWs) to get vaccinated.

Israel (population 9.136 million) was among the first countries that started a national vaccination campaign a short time after the BNT162b2 COVID-19 mRNA vaccine (Pfizer-BioNTech) received emergency use authorization from the United States Food and Drug Administration (FDA) [2]. The majority of those vaccinated in Israel have received BNT162b2. The mortality rate of confirmed COVID-19 cases in Israel was among the lowest in the world, and it remained so throughout the pandemic [3].

All permanent residents in Israel are covered by national health insurance under the National Health Insurance Law 1994, which is financed mainly through income-related tax revenues. Each resident is insured by one of the four health maintenance organizations (HMOs) and is eligible to receive the health services provided in the national list of health services (the health basket) [4]. The primary responsibility for vaccinating the general population was assigned to these HMOs. Each HMO was responsible for vaccinating its own members and able to quickly rent or otherwise access large facilities suitable for vaccinating large numbers of people [2]. Steps were also taken to vaccinate several special populations who do not have Israeli residency [5]. The success of Israel’s vaccination program was, among other things, attributed to the nationwide accessibility of the vaccine.

During December 2021, with the emergence of the Omicron variant in Israel, the prevalence of confirmed infection cases rose sharply. In an effort to address the challenges presented by this variant and to reduce the load on the healthcare system, on 2 January 2022, Israeli authorities were the first in the world to approve the administration of a fourth vaccine dose (second booster) for individuals aged 60 and over, high-risk populations, and HCWs who had received a first booster at least 4 months earlier. The major argument for this decision was that the expected benefit of reduced transmission and disease from a booster dose would outweigh the potential risks [6]. This decision was highly controversial due to the lack of evidence available at the time [7]. Other countries, such as the United Kingdom, had also started to offer fourth-dose booster vaccines to their populations at that time [8]. In March 2022, the FDA authorized a second booster dose of COVID-19 vaccines for older people and certain immunocompromised individuals [9]. By the end of June 2022, 814,791 Israelis had received the fourth vaccine dose [10].

The reasons why people choose not to get vaccinated are complex. In 2019, the World Health Organization (WHO) listed vaccine hesitancy among the top 10 threats to global health. The key reasons identified as underlying hesitancy were complacency, inconvenience in accessing vaccines, and lack of confidence [11]. Reasons cited for COVID-19 vaccine hesitancy included suspicion toward the short time it took to develop the COVID-19 vaccines and therefore also concerns about its claimed safety and effectiveness [12]. In contrast, the effectiveness and minimal adverse effects of the vaccine were the most common predictors of global acceptance of COVID-19 [13].

Attitudes of HCWs toward vaccines, and specifically the COVID-19 vaccine are extremely important for increasing vaccination coverage. HCWs are the most trusted advisors and influencers of vaccination decisions [11], and thus play a key role within vaccination programs [14]. However, HCWs have demonstrated vaccine hesitancy towards vaccinations, including COVID-19 vaccines. In Israel, and in other developed countries, health authorities’ recommendations for the vaccination of HCWs were met with only partial compliance [15]. For example, the seasonal influenza vaccination rate among HCWs is generally unsatisfactory [16,17]. Studies have shown that HCWs’ vaccination barriers resemble those of the general public and include concerns about adverse effects and vaccination novelty, lack of faith in the vaccine’s efficacy and in the severity of the disease [18], mistrust of government and institutions, and feeling that personal rights are being infringed upon [19].

We conducted a cross-sectional study at the beginning of the vaccination campaign of the fourth COVID-19 vaccine dose (second booster) to evaluate the attitudes of HCWs towards the fourth dose and the willingness to get vaccinated with it. At the time of the study, the FDA had not yet approved the second booster vaccine, providing a unique opportunity to explore this issue in Israel.

## 2. Materials and Methods

### 2.1. Setting and Participants

This cross-sectional survey included a sample of three groups of HCWs: (1) a group of HCWs working at a general public hospital located in central Israel, (2) medical interns working at the same hospital, and (3) HCWs who were health systems management students in an academic program at the time of the study. These HCWs were also asked to pass on the link of the survey to other HCWs.

### 2.2. Study Tool

A self-administered electronic questionnaire was constructed based on a literature review on hesitation toward COVID-19 vaccination among HCWs in Israel [20] and worldwide towards a booster dose among the general public [21] and HCWs [22,23]. After the questionnaire was constructed, it was tested on a convenience sample of 15 HCWs. Following their comments, some of the questions were revised.

The final questionnaire (Supplementary File S1) consisted of 4 sections: The first section evaluated risk perception toward the fourth dose of the COVID-19 vaccine. In the second section, respondents were asked about their COVID-19 vaccination history (whether they had received at least two doses of the COVID-19 vaccine, as well as the third dose (first booster)). They were also asked whether they are willing to receive the fourth vaccine dose. This question had the following answer options: “Yes, I already got the fourth dose”; Yes, I plan to get the fourth dose; No, I will wait to review safety data; No, I am not sure; “No, I do not plan to get the fourth dose”. Respondents who replied that they were not vaccinated or willing to get vaccinated were asked about the reasons for this (multiple answers). The next three questions in this section measured the willingness of the participants to vaccinate their children, acceptance of a hypothetical yearly booster dose, and having adverse events after prior vaccination. The third section examined perceptions of the fourth COVID-19 vaccine dose: safety, effectiveness, benefits, and importance of freedom of choice for HCWs in deciding to get vaccinated, trust in the MOH recommendations, and trust in the doctors’ recommendations. The fourth section included 7 questions on the participants’ sociodemographic characteristics (gender, age, marital status, number of children, profession, spoken language, research work). Additionally, the respondents rated their health status and the extent to which they regularly perform screening tests. Most of the questions were on a 10-point Likert scale from 1 (to a very small extent) to 10 (to a very large extent).

### 2.3. Data Collection

The survey was disseminated in January 2022 by email and social media (WhatsApp, Version 22.13.74) using a distribution list and by email using the snowball method. The questionnaires were completed anonymously.

### 2.4. Statistical Analysis

Statistical analysis was performed using SPSS version 26 (IBM Corporation, Armonk, NY, USA). The respondents were divided into two groups: those who reported that they were vaccinated with the fourth dose, or were willing to get it, were identified as “vaccinated HCWs”. Those who did not accept the fourth dose or responded that were waiting or unsure were identified as “having negative perception toward the fourth vaccine dose” (NegP4D).

Collected data were analyzed by descriptive statistics. Categorical variables were described using frequency and percentage and compared by chi-squared test. Continuous variables were described using mean and standard deviation and compared using an independent *t*-test.

To create a “total benefit” variable, a mean value was calculated for four questions on the booster’s benefits: perceived benefit, effectiveness, protection from severe illness, and booster’s advantages (Cronbach’s alpha = 0.81 for the internal consistency among the variables contributing to the perceived benefit). A “total risk” variable was calculated for 3 questions on the booster’s risk: risk perception, severity of health risk involved with getting vaccinated with the fourth dose, and perceived safety (Cronbach’s alpha = 0.86).

A hierarchical logistic regression model was applied for predicting the willingness of HCWs to get the fourth vaccine dose. Values of *p* < 0.05 were considered statistically significant.

## 3. Results

### 3.1. Sociodemographic Characteristics of the Study Population

The study population included 124 HCWs: 76 physicians (61.8%), 18 nurses (14.6%), and 29 other healthcare professionals (23.6%). The respondents’ sociodemographic and professional characteristics are shown in Table 1. The respondents’ mean age was 37.18 ± 7.8 years (range: 26–60). Most of the respondents were women (62.9%), were married or lived with a partner (77.4%), spoke Hebrew (84.7%), and were engaged in research only to a small extent (4.16 ± 2.8).

The mean perceived health status of the study population was “good” (8.94 ± 1.35 out of a possible 10). Most respondents (88.7%) reported being vaccinated against COVID-19 with two doses and the first booster (Table 1). Almost two-thirds of the respondents (76/124, 61.3%) did not get the fourth vaccine dose: 12.1% were waiting to review more data, 24.2% were not sure if they would get the fourth dose, and 25% did not plan to get it.

To identify the characteristics of vaccinated and NegP4D, the characteristics of the two groups were compared. Most women (69.2%) were not willing to get the fourth vaccine dose compared to 47.8% of men (0.022). Analysis by profession showed that 53.9% of physicians were unwilling to get vaccinated compared to 83.3% of nurses (*p* = 0.046) and 69% of HCWs with other professions.

**Figure 1:**
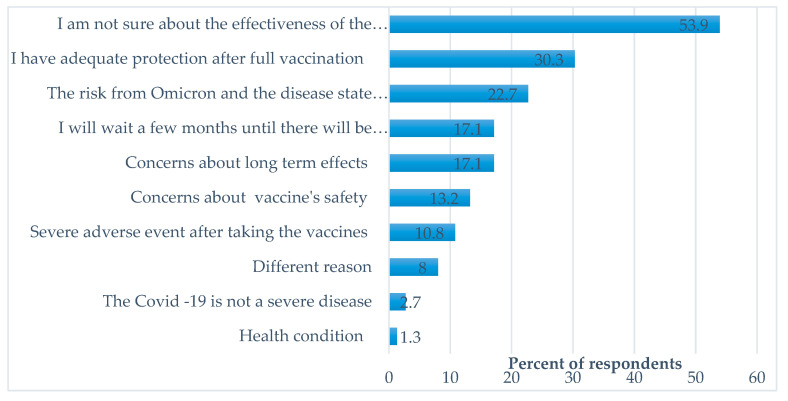
Reasons for unwillingness to get the fourth COVID-19 vaccine dose.

The reasons for unwillingness to get vaccinated with the booster dose are depicted in Figure 1. The most frequent reason was lack of faith in the effectiveness of the booster dose (53.9%); 30.3% of the respondents believed that they have adequate protection after two vaccine doses and therefore do not need a booster dose, and 22.7% believed that the risk from COVID-19 and the Omicron variant are not significant, and therefore the booster dose is not necessary. Waiting a few months until there will be sufficient knowledge about safety and effectiveness influenced 17.1% of HCWs. Concerns about long-term vaccination effects (17.1%) and the vaccine’s safety (13.2%) were also influential reasons.

### 3.2. Attitudes toward the Fourth COVID-19 Vaccine Dose (Second Booster)

Examination of the perceptions of HCWs towards the fourth COVID-19 vaccine dose showed that all variables were statistically significantly different between the vaccinated and Neg4PD groups (Table 2). The Neg4PD group perceived the fourth vaccine dose as having a higher risk compared to the “vaccinated” group (mean: 4.2 ± 2.2 vs. 2.2 ± 1.5, *p* < 0.001). The perceived health risk severity of getting the fourth vaccine dose was also rated higher by the Neg4PD group compared to the “vaccinated” group (*p* < 0.001). On average Neg4PD group perceived the freedom to choose whether to get vaccinated as greater than “vaccinated” HCWs (7.6 ± 3 vs. 6.0 ± 3.3, *p* = 0.005). All other variables related to the perceived benefit of the booster, its advantages, its perceived safety, protection from severe COVID-19 illness, and the perceived extent of knowledge that science has about the risk related to the booster received higher scores from “vaccinated” HCWs compared with the Neg4PD HCWs (*p* < 0.001). “Vaccinated” HCWs also gave higher scores to the perceived novelty of the health risk and to the subjective knowledge compared to Neg4PD HCWs (*p* = 0.014). Additionally, the “vaccinated” group rated trust in the MOH and in healthcare providers higher than Neg4PD HCWs (*p* < 0.001).

### 3.3. Attitudes toward Vaccination of Children, Acceptance of a Hypothetical Yearly Booster Vaccine, and Reported Adverse Effects following a Prior COVID-19 Vaccination

**Figure 2:**
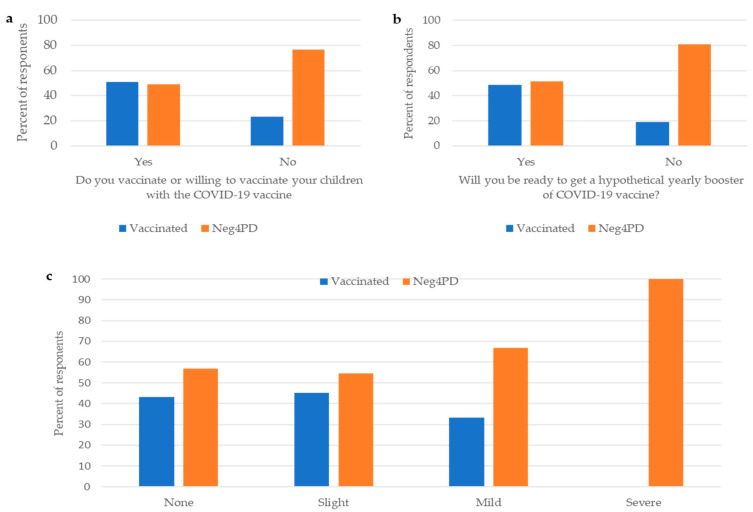
Attitudes of HCWs toward (**a**) vaccination of children; *p* = 0.04 for the difference between vaccinated and Neg4PD HCWs who responded that they would/would not vaccinate their children, (**b**) acceptance of a hypothetical annual booster vaccine; *p* = 0.002 for the difference between vaccinated and Neg4PD HCWs who responded that they would/would not get vaccinated with a hypothetical annual vaccine, and (**c**) reported adverse effects following prior COVID-19 vaccination; *p* = 0.037 for the difference between vaccinated and Neg4PD HCWs in the severity of adverse effects following prior vaccination with COVID-19 mRNA vaccine. NegP4D = having negative perception toward the fourth vaccine dose.

The willingness to vaccinate their own children, the acceptance of hypothetical yearly booster vaccine, and having adverse effects after getting a vaccine were associated with respondents’ willingness to get the fourth vaccine dose and were statistically significantly different between the two groups (Figure 2).

Over half of the respondents (50.9%) who reported that they vaccinate or intend to vaccinate their children were “vaccinated” HCWs compared to 23.5% of Neg4PD (*p* = 0.046). Most Neg4PD HCWs reported that they would not choose to get vaccinated with an annual COVID-19 vaccine should it become available compared to 19% of “vaccinated” HCWs (*p* = 0.002). Finally, all of the respondents who reported having severe adverse effects after a prior COVID-19 vaccine were Neg4PD HCWs. Among respondents who reported they did not have adverse effects at all, 56.8% were Neg4PD and 43.2% were “vaccinated”. 66.7% of the “not vaccinated” HCWs reported having mild adverse effects. (*p* = 0.037).

### 3.4. Perception of the Risk and Benefit of the Fourth Dose Significantly Predict HCWs’ Willingness to Get the Fourth Dose of the COVID-19 Vaccine

A hierarchical logistic regression model was applied for predicting the willingness of HCWs to get vaccinated with the fourth COVID-19 vaccine dose (Table 3).

Gender and profession were added to the first step, followed by prior vaccination with the COVID-19 vaccine (second step), perceived benefit (third step), and perceived risk (fourth step). Only the perceived risk and perceived benefit of the vaccine made a unique statistically significant contribution to the model (X^2^(6) = 66.573, * *p* < 0.001, ** *p* < 0.05).

## 4. Discussion

The evolving virus variants of SARS-CoV-2, such as the Omicron variant, which caused widespread morbidity, and the waning immunity to the virus, including that observed in vaccinated individuals, have led to the implementation of COVID-19 vaccine booster doses, which may become seasonal. HCW vaccination compliance is a challenge for health leaders as they are the most trusted advisors and influencers for vaccination decisions [24] and thus hesitant HCWs pose a risk of lowering intervention uptake among the general population [25].

This cross-sectional study, which was conducted shortly after the Israel MOH had recommended HCWs to get vaccinated with the fourth vaccine dose, presents a snapshot of a point in time when available information on the effectiveness and safety of the vaccine’s booster was scarce. As such uncertainty naturally affects the decision to get vaccinated, it presented a unique opportunity to gather viewpoints from HCWs in a country with a high vaccination rate (by January 2022, 80% of the eligible population had received two doses of COVID-19 vaccine plus a booster dose).

### 4.1. Reasons for Hesitation to Receive a Fourth Dose of COVID-19 Vaccine

Our findings showed that at the time of the study, most of the respondents were not vaccinated with the fourth vaccine dose, and the most frequent concerns regarding the vaccine were its efficacy, benefit, and necessity. These may be explained by the time period and the specific virus variants at the time the booster was authorized in Israel. The study was conducted shortly after the Israel MOH had recommended HCWs to get vaccinated with the fourth vaccine (second booster); the FDA had not yet approved this booster, and available information on its effectiveness and safety was scarce. Compared to previous vaccine doses, the MOH’s recommendation was indecisive and ambiguous due to the lack of evidence available at the time [7]. This has probably contributed to the observation that more than half of the respondents were concerned about its effectiveness. The multidose regimen, as well as the booster dose, may have been perceived as an indication of the vaccine’s ineffectiveness [19,23]. In a study among HCWs in the Czech Republic, the perceived effectiveness of the COVID-19 booster dose was a significant predictor of acceptance [22].

Concerns about safety and adverse effects of the vaccine were one of the most frequent reasons for COVID-19 vaccine hesitancy among HCWs [22,23,26,27]. This was also observed in the general public around the world [20,28]. Adverse effects experienced after the previous (primer) doses were mentioned as a reason for accepting or rejecting a booster dose.

Beyond the uncertainty about its benefit or safety due to the lack of real-world evidence [19], its necessity was also questioned. At the time of the fourth dose’s approval, there was an indication that the Omicron variant generally causes less severe disease than prior variants [29], and HCWs did not see the same number of severely ill patients as was in prior cases. In addition, the World Health Organization’s position was that a fourth vaccine dose was not required to maintain immunity, except for people who are immunocompromised [30]. These views were reflected in the answers of the HCWs who did not plan to get vaccinated and corroborated the findings of other studies that examined attitudes on COVID-19 vaccination and booster doses among HCWs [19]. In a survey of the general population in Poland, lack of faith in the booster’s effectiveness or the belief that the basic vaccination schedule ensures an adequate level of protection were reasons for refusing to take the booster [21].

### 4.2. Demographic Factors

Gender and profession were significantly associated with willingness to take the fourth dose. Most women were unwilling to get the fourth vaccine dose compared to men. About half of physicians (53.9%) were unwilling to get vaccinated compared to 83.3% of nurses and 69% of other HCW professions. These findings are consistent with the findings of other studies, which show that vaccination intention varies with gender and profession [19,20,22,31,32]. It was suggested that psychological gender differences and the cautious approach of women to acceptance of innovative medical technologies underlie these differences, misunderstanding of vaccines, nurses being mainly women, having lower risk perception than doctors, and less fear of COVID-19 [19,33,34,35].

### 4.3. Perceptions of the Fourth Dose

We identified significantly different perceptions and attitudes regarding the fourth vaccine dose between vaccinated HCWs and HCWs who did not intend to get vaccinated. As expected, the perceived risk and perceived severity of the health risk associated with getting the fourth vaccine dose were rated higher by those who stated that they would not get vaccinated. The perceived risk of a vaccine is correlated with the willingness to get vaccinated [36]. This group also gave higher scores than the “vaccinated” group on the importance of having the freedom to choose whether to get vaccinated. In contrast, HCWs who were vaccinated with the fourth dose, or those who were planning to get vaccinated, gave higher scores to the perceived benefit of the booster, its advantages, its perceived safety, its ability to protect from severe illness, and perceived extent of scientific information about the risk associated with the booster. Similar results were found among HCWs in the United States where HCWs willing to be vaccinated perceived greater benefits and fewer barriers to vaccination against COVID-19 compared to vaccine-hesitant HCWs [37]. Beyond that and most importantly, the logistic regression model revealed that perception of the fourth dose’s benefits and risk significantly predict the willingness of HCWs to get vaccinated.

Vaccine hesitancy changes over time because of the public’s ever-changing perception of the risk of COVID-19 and information related to vaccination safety and efficacy [38,39]. At the beginning of the COVID-19 vaccination campaign, the most prevalent concerns of physicians were the vaccine’s fast development and approval, safety, and effectiveness [18]. Although some of these concerns may have subsided by the time the fourth vaccine dose was approved, the rollout of booster doses, vaccine and pandemic fatigue, adverse effects from prior doses, breakthrough infections, and lower perceived risk of the disease with the declining cases of infections could lead to questioning by the public about the utility and need of the boosters, their effectiveness, and whether boosters are a viable solution for pandemic control [40].

This study demonstrates that HCWs’ willingness to get vaccinated depends mostly on their perception of the vaccine as beneficial, necessary, and not carrying risks. Given that most of the HCWs were fully vaccinated, it is reasonable to assume that they have some degree of confidence in the vaccine, but at the same time, pandemic fatigue or lower perceived risk of the disease could lead to their questioning the utility, effectiveness, and need for the booster dose [41]. Studies of special population groups, including HCWs, have also found an association between vaccine confidence and booster dose willingness. Risk–benefit ratio estimation was also a significant and robust predictor of booster acceptance among HCWs as well [22].

To date, data from Israel have shown the effectiveness of the fourth COVID-19 vaccine dose. They boost cellular and humoral immunity. Rates of confirmed SARS-CoV-2 infection and severe COVID-19 were lower after a fourth dose of the BNT162b2 vaccine than after only three doses [42,43,44]. Therefore, providing information on the risk–benefit ratio of vaccination is of paramount importance [31]. Along with efforts to reduce barriers and the perceived risk of the booster doses.

The various concerns and perceptions expressed by HCWs with regard to the fourth vaccine dose highlight that HCWs have a unique position. On the one hand, they are an extension of healthcare systems, and on the other hand, they are part of the public as complex individuals with personal lives, values, and opinions that impact their attitudes, hesitancy, and behaviors towards disease prevention interventions [25].

### 4.4. Trust, Willingness to Vaccinate Children, and the Acceptance of Hypothetical Yearly Booster Vaccine

Other factors that have been found to be associated with the acceptance of the fourth vaccine dose and characterize the difference between HCWs who are vaccinated and those who do not intend to get vaccinated are the willingness to vaccinate their own children, willingness to take a hypothetical annual booster vaccine, and the level of trust in the MOH and in healthcare providers. Studies have shown that HCWs who are hesitant to get vaccinated, including booster dose, have lower levels of trust in authorities and the healthcare system [19,23,45].

The willingness to vaccinate their own children and the willingness to get an annual hypothetical booster dose were associated with willingness to take the fourth dose. These findings are in line with those of another study in which the acceptance of a hypothetical annual booster vaccine was higher among unhesitant HCWs [23]. It was noted that prior refusal to get vaccinated against other illnesses, such as influenza, is associated with COVID-19 vaccine hesitancy; therefore, initial COVID-19 vaccine vaccination may be a predictor of booster hesitancy [23].

### 4.5. Limitations

The study was performed shortly after the Israel MOH recommended HCWs to get vaccinated with the fourth vaccine while the Omicron variant spread across the country. Therefore, our findings provide a view of a particular point in time and should be interpreted in relation to its timing. The accumulating information on the vaccine’s safety and effectiveness at the time of the study may have affected the respondents’ attitudes toward it. Second, due to the urgency of designing the study at the beginning of the vaccination campaign, a convenience sample of HCWs was used. The small sample and low response rate of HCWs could be explained by the high workload in hospitals, high morbidity, and burnout of staff. Other studies have also reported low response rates among HCWs. The percentage of women among the respondents was relatively high; however, this represents the population of HCWs around the world [46,47].

## 5. Conclusions

The low acceptance of the fourth COVID-19 vaccine dose among HCWs can affect the health system and have broad consequences. Insufficient vaccination uptake can lead to increased COVID-19 infections, less available health staff, and increased workload in hospitals, and thus reduce the capacity of the health system to adequately respond to the pandemic [48]. HCWs play a key role within COVID-19 vaccination programs, as they are the trusted advisors and influencers of vaccination decisions. Studies have shown that vaccinated HCWs are more likely to recommend vaccines to friends, family, and their patients [23]. Thus, hesitant HCWs pose a risk of lowering intervention uptake among the general population [25]. The health system should be more tolerant of their concerns and address them through communication [49].

Perception of the fourth dose benefit and risk significantly predict the willingness of HCWs to receive the fourth COVID-19 vaccine dose. These findings are extremely relevant for booster vaccination campaigns, indicating a need for ongoing relevant communication regarding the benefits of the fourth vaccine dose and its necessity, along with efforts to reduce barriers and perceived risk of the booster doses.

The MOH’s recommendation for a fourth vaccine dose, together with communicating the need for high vaccination uptake in order to halt viral spread, and the personal example set by HCWs who were vaccinated with the fourth dose, can be part of a vaccination campaign.

The findings of the study may also reflect the attitudes of the general public and can also be used for implementing other new vaccine technologies. As long as the scientific knowledge about a vaccine is incomplete, and as information and perceptions of the vaccine change over time, our findings could help policy makers in developing strategies to expand the acceptance and coverage of the COVID-19 booster doses and other vaccines among HCWs and the general public.

## Figures and Tables

**Table 1:** Comparison of the sociodemographic and professional characteristics of vaccinated HCWs and those unwilling to get vaccinated.

Characteristic	NegP4D N = 76	Vaccinated N = 48	All N = 124	*p* Value
Age, years, Mean (SD)	36.3 (7.35)	38.5 (8.35)	37.18(7.8)	0.129
Gender, n (%)				0.022
Male	22 (47.8%)	24 (54.2%)	46 (37.09%)	
Female	54 (69.2%)	24 (30.8%)	78 (62.9%)	
Marital status, n (%)				0.118
Married/living with a partner	57 (59.4%)	39 (40%)	96 (77.4%)	
Single	18 (75%)	6 (25%)	24 (19.4%)	
Divorced/widowed	1 (25%)	3 (75%)	4 (3.22%)	
Number of children, mean (SD)	1.68 (1.87)	1.64 (1.57)	1.66 (1.75)	0.906
* Profession				0.046
Physician	41 (53.9%)	35 (46.1%)	76 (61.8%)	
Nurse	15 (83.3%)	3 (16.7%)	18 (14.6%)	
Other healthcare professional	20 (69%)	9 (31%)	29 (23.6%)	
Perceived health status *, mean (SD)	9.08 (1.2)	8.72 (1.5)	8.94 (1.35)	0.153
Undergoes screening tests regularly **, mean (SD)	6.3 (2.8)	6 (2.9)	6.2 (2.8)	0.662
COVID-19 vaccination history, n (%)				
1 + 2 dose only	10 (83.3%)	2 (16.7%)	12 (9.7 %)	0.123
1 + 2 + 3 doses	64 (58.2%)	46 (41.8%)	110 (88.7%)	
Engaged in research **, mean (SD)	4.21 (2.94)	4.09 (2.59)	4.16 (2.8)	0.815

NegP4D = having negative perception toward the fourth vaccine dose; SD = standard deviation. ** On a scale of 1 to 10 * One respondent did not report his profession.

**Table 2:** Respondents’ mean scores in the study questionnaires by study population.

	Scale	NegP4D N = 76 Mean (SD)	Vaccinated N = 48 Mean (SD)	All N = 124 Mean (SD)	*p* Value
Risk perception of using the fourth COVID-19 vaccine dose	1–10	4.2 (2.7)	2.2 (1.5)	3.4 (2.5)	<0.001
Perceived benefit	1–10	3.7(1.9)	6.2 (2.1)	4.7 (2.3)	<0.001
Subjective knowledge	1–10	3.9(2.5)	5.1(2.7)	4.4 (2.6)	0.014
Novelty of health risk	1–10	3.8(2.4)	4.9(2.6)	4.2 (2.5)	0.014
Severity of health risk involved with being vaccinated with the fourth dose	1–10	4.3(2.3)	2.6(1.3)	3.6 (2.1)	<0.001
Perceived extent of knowledge that science has about the safety of the fourth dose	1–10	4.1 (2.2)	5.4 (2.5)	4.6 (2.4)	<0.001
Booster safety	1–10	5.8 (2.6)	8.1 (1.8)	6.7 (2.6)	<0.001
Booster effectiveness Protection from severe illness	1–10 1–10	4.3 (2.5) 5.4 (2.6)	5.8 (2.3) 7.6 (2)	4.9 (2.5) 6.2 (2.6)	0.001 <0.001
More advantages than disadvantages Trust in the Ministry of Health	1–10 1–10	4 (2.1) 4.1(2.4)	7.7 (1.7) 6.9(2.3)	5.4 (2.7) 5.2 (2.7)	<0.001 <0.001
Trust in doctors Freedom of choice	1–10 1–10	4.57 (2.5) 7.67 (3)	7.7 (1.9) 6.02 (3.3)	5.7 (2.8) 7 (3.2)	<0.001 0.005

NegP4D = having negative perception toward the fourth vaccine dose; SD = standard deviation.

**Table 3:** Hierarchical logistic regression to determine the predictors of willingness to get vaccinated with fourth COVID-19 vaccine dose.

Variable	b(se)	Model 1 OR [95% CI]	b(se)	Model 2 OR [95% CI]	b(se)	Model 3 OR [95% CI]	b(se)	Model 4 OR [95% CI]
Sociodemographic								
Gender	0.58(0.45)	1.79 [0.73, 4.36]	0.59 (0.45)	1.80 [0.73, 4.43]	0.55 (0.56)	1.74 [0.58, 5.23]	0.3 (0.57)	1.35 [0.43, 4.16]
Profession doctor	Ref.	Ref.	Ref.	Ref.	Ref.	Ref.	Ref.	Ref.
Profession nurse (Ref. doctor)	−1.12 (0.7)	0.32 [0.08, 1.29]	−1.10 (0.70)	0.33 [0.08, 1.33]	−0.80 (0.83)	0.44 [0.08, 2.27]	−0.56 (0.84)	0.56 [0.11, 2.95]
Profession health care (Ref. doctor)	−0.25 (0.53)	0.77 [0.27, 2.21]	−0.15 (0.54)	0.86 [0.29, 2.50]	−0.22 (0.72)	0.79 [0.19, 3.29]	−0.135 (0.78)	0.87 [0.18, 4.03]
Health Related								
Prior COVID-19 vaccination			1.17 (0.81)	3.23 [0.65, 16.04]	1.92 (1.16)	6.82 [0.69, 66.849]	1.93 (1.22)	6.91 [0.62, 76.2]
Perceptions								
Total benefit					0.95 (0.18)	2.58 [1.81, 3.69]*	0.84 (0.18)	2.33 [1.62, 3.36] *
Total risk							−0.47 (0.19)	0.62 [0.43, 0.90] **
Constant	−0.49 (0.35)	0.612	−1.60 (0.86)	0.20	−7.61 (1.76)	0.00	−5.51 (1.87)	0.004 *
Model χ^2^		7.4		9.90		59.34, *p* < 0.001		66.57, *p* < 0.01
Step χ^2^		7.42		2.47		49.44, *p* < 0.001		7.22, *p* < 0.01
Naglekerke R2		0.08		0.10		0.52		0.57

## Data Availability

Data available upon request.

## Supplementary Materials

The following supporting information can be downloaded at: https://www.mdpi.com/article/10.3390/vaccines11020385/s1, questionnaire: Attitudes of healthcare workers in Israel towards the fourth dose of COVID-19 vaccine.
