# Supplementary material for: Attitudes of Healthcare Workers in Israel towards the Fourth Dose of COVID-19 Vaccine
Source: Vaccines (Basel). 2023 Feb 7;11(2):385. doi: 10.3390/vaccines11020385 (PMC9966952; doi:10.3390/vaccines11020385)
Supplement: Supplementary file 1 [file vaccines-11-00385-s001.zip › vaccines-2147344-supplementary.pdf]

Attitudes of healthcare workers in Israel towards the fourth dose of Covid-19 vaccine

In this questionnaire you will be asked about the fourth dose of covid-19 vaccine.

1. Please rate, on a scale of 1 (to a very small extent) to 10 (to a very large extent), to what extent you think the use of the fourth dose of COVID-19 vaccine poses a health risk for you.

Fourth dose Covid-19 vaccine	1	2	3	4	5	6	7	8	9	10

Please rate on a scale of 1 (to a very small extent) to 10 (to a very large extent):

	to a very small extent									to a very large extent
	1	2	3	4	5	6	7	8	9	10
2.To what extent is the use of the fourth dose COVID-19 vaccine important for dealing with the virus?										
3.To what extent do you have knowledge about the possible health risk from the use of the fourth dose Covid-19 vaccine?										
4.To what extent is the health risk from the vaccine known and familiar?										
5.If there is a health risk in the fourth dose Covid-19 vaccine how serious is it?										
6.To what extent do you think science has enough knowledge about the safety of using the fourth dose of Covid-19 vaccine?										

7. Did you receive the Covid-19 vaccine?
1) No 2) Yes- Only the first dose. 3) Yes- Two doses. 4) Yes- Two doses as well as the third dose (first booster).
8. Did you receive the fourth dose of Covid-19 vaccine or you are willing to receive it? "

1) Yes, I already got the fourth dose. 2) Yes, I plan to get the fourth dose 3) No, I will wait to review safety data. 4) No, I am not sure. 5) No, I do not plan to get the fourth dose.

9. If you are not willing to get vaccinated with the fourth dose soon, what is the reason for this? (multiple answers).

I had severe adverse event after taking the vaccines

Concerns about long term effects

I am not sure about the effectiveness of the vaccine

Concerns about vaccine's safety

I have adequate protection after full vaccination

The risk from the Omicron variant and the Covid-19 disease are not significant, and therefore the booster dose is not necessary

I will wait a few months until there will be sufficient knowledge about safety and effectiveness of the vaccine.

Health condition

Other

10. Do you vaccinate or willing to vaccinate your children with the Covid-19 vaccine?

1) Yes 2) No 3) I have no children.

11. Will you be ready to get a hypothetical yearly booster of Covid-19 vaccine?

1) Yes 2) No

12. Did you have adverse events after prior vaccination?

1) No 2) Yes- Slight 3) Yes- Mild 4) Yes- Severe

To what extent do you agree with the following sentences: 1 (I strongly disagree) to 10 (I strongly agree):

13. The fourth dose of Covid-19 vaccine is as safe as prior Covid-19 vaccines.

|1| |2| |3| |4| |5| |6| |7| |8| |9| |10|

14. The fourth dose is effective in preventing infection with the disease

|1| |2| |3| |4| |5| |6| |7| |8| |9| |10|

15. The fourth dose can protect me from a serious illness

|1| |2| |3| |4| |5| |6| |7| |8| |9| |10|

16. The advantages of receiving the fourth dose vaccine outweigh the disadvantages

|1| |2| |3| |4| |5| |6| |7| |8| |9| |10|

17. It is importance of give HCWs freedom of choice in deciding to get vaccinated

|1| |2| |3| |4| |5| |6| |7| |8| |9| |10|

18. I trust the MOH recommendations to get the Covid-19 fourth dose vaccine .and trust in the doctors' recommendations

|1| |2| |3| |4| |5| |6| |7| |8| |9| |10|

19. I trust in the doctors' recommendations to get the Covid-19 fourth dose vaccine.

|1| |2| |3| |4| |5| |6| |7| |8| |9| |10|

Sociodemographic questions

20. Age

21. Gender

22. Marital status

Married/living with a partner/ Single/divorced/widowed

23. Number of children

24. Profession

Physician/Nurse/Other healthcare professional

25. To what extent do you engage in research in your work? (to a very small extent) to 10 (to a very large extent)

|1| |2| |3| |4| |5| |6| |7| |8| |9| |10|

26. What is your spoken language?

Hebrew/ Arabic/ Russian/ Amharic/ Other

27. Please rate your health on a scale of 1 (not good) to 10 (very good).

|1| |2| |3| |4| |5| |6| |7| |8| |9| |10|

28. To what extent are you undergoing regular health screening tests? (to a very small extent) to 10 (to a very large extent)

|1| |2| |3| |4| |5| |6| |7| |8| |9| |10|
